# Infection control in home care.

**DOI:** 10.3201/eid0702.010211

**Published:** 2001

**Authors:** E Rhinehart

**Affiliations:** AIG Consultants, Inc., Atlanta, Georgia, USA. emily.rhinehart@aig.com

## Abstract

Although home care has expanded in scope and intensity in the United States in the past decade, infection surveillance, prevention, and control efforts have lagged behind. Valid and reliable definitions and methods for surveillance are needed. Prevention and control efforts are largely based upon acute-care practices, many of which may be unnecessary, impractical, and expensive in a home setting. Infectious disease control principles should form the basis of training home- care providers to assess infection risk and develop prevention strategies.


### Infection Control in Home Care

**Emily Rhinehart**
AIG Consultants, Inc., Atlanta, Georgia, USA

Although home care has expanded in scope and intensity in the United States in the past decade, infection surveillance, prevention, and control efforts have lagged behind. Valid and reliable definitions and methods for surveillance are needed. Prevention and control efforts are largely based upon acute-care practices, many of which may be unnecessary, impractical, and expensive in a home setting. Infectious disease control principles should form the basis of training home-care providers to assess infection risk and develop prevention strategies.

Efforts to decrease length of hospital stay and shift care to ambulatory settings, as well as patient and family preference to receive care at home, have contributed to the substantial growth of home care in the past decade. As life expectancy in the U.S. population continues to increase and patients with chronic illnesses live longer, home care will continue to expand.

Home care has also broadened in type and scope in the past decade. Most patients are elderly and have chronic conditions requiring skilled nurses and aides. High-tech home care is provided to patients of all ages and may include home infusion therapy, tracheotomy care and ventilator support, dialysis, and other highly invasive procedures. In addition, home-care nurses provide assessment, education, and support to post-acute-care patients who might have spent several additional days in the hospital but are now discharged to cut costs. This category of patient may include postoperative patients, postpartum mothers and their newborns, and patients with acute medical conditions such as newly diagnosed diabetes and recent strokes.

In the United States, 9,655 agencies (1998 data) (1) provide home care to patients. Infection control and health-care epidemiology have not kept up with the needs of the home-care providers or their patients. As this segment continues to expand and services provided in the home increase, the infection control community must address the risks and needs of home care.

### Infection Surveillance, Prevention, and Control in Home Care

Infection surveillance, prevention, and control have constituted a discipline that has been acute-care based and oriented for the past 40 years. However, as the health-care system continues to shift delivery of care from hospitals to other settings, surveillance, prevention, and control programs must respond. Since efforts to measure the incidence of home-care acquired infections, study the associated risk factors, and adapt prevention and control measures for home care are nascent, available studies provide minimal information and little guidance. A few articles have appeared in non-U.S. publications. Overall, the literature is sparse, but expanding slowly (2-22).

### Systems of Surveillance: Definitions and Methods

Without valid data on the incidence of home-care acquired infection and analysis of risk factors, developing control efforts is difficult. Thus, initial resources must be directed toward developing measurement systems. Definitions and methods for the surveillance of nosocomial infection cannot be readily applied to home care. First, definitions, such as those developed by the Centers for Disease Control and Prevention's (CDC) National Nosocomial Infection Surveillance (NNIS) system (23), rely heavily on laboratory data, including cultures and serologic tests. In home care, the diagnosis of infection for clinical purposes is frequently made on an empiric basis with substantial reliance upon physical signs and symptoms. In fact, physicians routinely rely on the assessment skills of home-care nurses and may not see a patient before making a presumptive diagnosis and writing prescriptions. The current reimbursement system does not support the use of cultures and laboratory tests used for hospitalized patients. For example, cultures are not routinely obtained to diagnose or confirm infections of the urinary tract, respiratory tract, or wound or skin sites. Cultures are more frequently obtained to confirm and appropriately treat bloodstream infection in patients undergoing home infusion therapy.

Definitions of home-care acquired infection developed for surveillance will need to rely more heavily on clinical signs and symptoms and tests that can be performed by the home-care nurse at the bedside (e.g., urine dipstick testing). A scheme that includes probable home-care acquired infection (i.e., clinical signs and symptoms of pneumonia) as well as definite home-care acquired infection (i.e., confirmed by chest X ray and sputum culture) may be considered. Once developed, definitions must be examined for validity, sensitivity, and specificity. However, methods to identify patients at risk and apply the definitions are also critical.

Surveillance methods routinely used in acute care, such as cultures and other laboratory tests, are not practical in home care (24) so other sources of information and methods of screening must be developed. In addition, a system that relies on a designated person(s) to review medical records and assess patients for infection, such as infection control

Address for correspondence: Emily Rhinehart, AIG Consultants, Inc., 1200 Abernathy Rd., NE, Suite 800, Atlanta, GA 30328, USA; fax: 770-399-4161; e-mail: emily.rhinehart@aig.com professionals do in hospitals, is impractical in home care because of the logistics of patients, staff, and medical records.

A more suitable approach is a two-tiered system, which relies on home-care nurses to identify and report patients with clinical signs and symptoms of infection and on an infection control nurse to review evidence and ascribe a definition (Table). Screening criteria for home-care nurses would include fever, new antibiotic order, purulent drainage from a wound, change in color or odor of urine, change in consistency or color of sputum, respiratory rales and rhonchi, and increased serum leukocytes. Once made aware of these patients, a designated nurse can review the evidence (e.g., clinical signs and symptoms, available laboratory data, nursing and physician progress notes) and apply the definition of home-care acquired infection. This approach should enhance both sensitivity (more nurses observing and reporting patients with clinical signs and symptoms of infection) and specificity (one nurse applying the definition of infection). The use of a single infection control nurse should also improve the reliability of data.

### What Is Needed

To achieve a system to measure and study the incidence and risks for home-care acquired infection, infection control must develop valid definitions for home-care acquired infection and practical methods for surveillance. These definitions and methods must be developed through a broad, national effort that includes participation by home-care professionals as well as infection control practitioners. These professionals must take a very practical approach to this endeavor and may have to forego rigid application of epidemiologic techniques for a more suitable surveillance system. The Association for Professionals in Infection Control and Epidemiology has recently published draft definitions for surveillance in home care (25). In parallel, home-care professionals must engage in learning the epidemiologic principles of surveillance systems (26) and apply or adapt them as faithfully as possible.

Once consensus is reached on definitions and methods and we describe the epidemiology of home-care acquired infections, we can study specific risk factors for infection. Home-care professionals need the assistance, support, and practical guidance of infection control professionals. Because of substantial financial challenges in home care, one nurse is often responsible for quality improvement, safety, risk management, and infection control. These professionals can apply and manage surveillance systems but will need substantial guidance and support in developing them.

Efforts to initiate surveillance systems do exist. The Missouri Home Care Alliance began a program in 1997 to develop definitions and collect data from home-care agencies in that and other states. With assistance from CDC's Hospital Infections Program, the alliance has made progress in developing a surveillance system and sharing data. The Florida Hospital Association also sponsored a surveillance project for hospital-based home-care agencies (6) in which they studied the incidence of urinary tract infections and central-line infections. The Arizona Association for Home Care also described its methods and results in a cooperative study to measure and compare rates of urinary tract infections (7). Similar efforts were undertaken in a collaborative effort to determine device-related rates of urinary tract and bloodstream infections in California, Kentucky, and Indiana (8). These studies provide initial descriptions of incidence of home-care acquired infections. Authors report catheter-related urinary tract infection rates

Table. Criteria for inclusion in definitions of home-care-acquired infection[a]

| Site of infection | Clinical data | Laboratory data |
|---|---|---|
| Catheter-related UTI[b] | Change in characteristics of urine, fever, pain | Elevated serum leukocytes, evidence of UTI in urinalysis, evidence of leukocytes in urine dipstick test, positive urine culture (>$10^5$ CFU of a single organism per mL urine) |
| Postoperative pneumonia | Change in character of sputum, decreased breath sounds, increase in rales and rhonchi, fever, shortness of breath, pain | Elevated serum leukocytes, sputum Gram-stained smear with evidence of respiratory infection, positive sputum culture, positive chest X ray |
| Catheter-related bloodstream infection | Fever with chills and rigors, redness, tenderness, or pain at insertion site, purulent drainage at site | Elevated serum leukocytes, positive blood culture, positive catheter culture (after catheter removal) |
| Skin and soft tissue infection | Pain, swelling, tenderness at site, inflammation and warmth, purulent drainage, fever | Gram-stain smear with leukocytes and organisms, positive culture, elevated serum leukocytes |
| Endometritis in postpartum patients | Uterine tenderness and abdominal pain, purulent vaginal drainage (lochia), foul-smelling lochia, fever | Positive Gram-stain smear of lochia, positive culture of lochia, remarkably elevated serum leukocytes |

[a]Source: Rhinehart E, Friedman M. Infection control in home care. Gaithersburg (MD): Aspen Publishing, Inc.;1999 (22).
[b]UTI = urinary tract infection.

of 2.8 per 1,000 catheter days (6) to 4.5 per 1,000 catheter days (8). Measures of intravenous catheter-related bloodstream infections range from 1.1 per 1,000 catheter days (8) to 4.2 per 10,000 catheter days (2). Data from these studies must be interpreted with caution, however, since surveillance in this area is in its initial stages and definitions and methods are not uniform. More studies are in progress, and eventually there will be consensus on such issues.

### Prevention and Control of Home-Care Acquired Infection

Even without reliable surveillance data, we know that infection prevention and control in home care is quite different from that in acute care. In acute care, a patient's risk for nosocomial infection is related not only to the severity of illness and exposure to invasive interventions and devices but also to environmental risks, including exposure to other patients and inanimate reservoirs of nosocomial pathogens. The home-care patient may have less clinical "acuity" (i.e., intensity or degree of care needed) but may have substantial host risk factors, including advanced age, chronic illness, or immunosuppression. Much of home care is provided by family members in a setting that is much less structured and controlled than the hospital environment. Plumbing, sanitation, and ventilation may be poor or absent. Nonetheless, basic principles of prevention and control can be adapted and applied with large doses of realistic risk assessment and common sense.

Because written resources for home-care practice are lacking, many home-care providers have adopted unnecesssary infection control practices to reduce risk for patients, including the ritual of nursing bag technique (i.e., placing a newspaper under the nursing bag), policies that require the routine disinfection of noncritical devices (e.g., stethoscopes and blood pressure cuffs) after every use, and procedures that require handwashing based on seemingly arbitrary criteria (e.g., upon entering the home). Some of these practices are not only unnecessary but also costly (e.g., routine changing of urinary drainage bags every 30 days).

Patient-care practices to reduce the risk for home-care acquired infection must be based on the basic science embodied in the chain of infection model. Actual risk and appropriate prevention and control strategies must be incorporated in recommendations for policy and procedure. Using this simple approach to determine actual risk and implement the appropriate prevention and control strategies will lead to more reasonable and less ritualistic practices for patient care and use of precautions to prevent the spread of infections to others. Infection control professionals should approach their responsibility to guide home-care providers by first addressing educational needs. Knowledge of infection control principles enables home-care providers to develop their own approaches to patient care and make decisions about infection risk and its reduction.

### Patient-Care Practices

Infection prevention strategies in home care should focus on home infusion therapy, urinary tract care, respiratory care, wound care, and enteral therapy. Most recommended practices on intravenous therapy (27) do not require adaptation for the home. However, in care involving other sites, the risk may be lower, allowing for adaptation of practices designed for hospitalized patients. For example, use of indwelling urinary catheters creates an inherent risk for infection. In the hospital, considerable efforts are exerted to maintain an intact, closed urinary drainage system (28); however, in home care the system is frequently interrupted when an ambulatory patient uses a leg bag. Drainage bags may also be disinfected in the home, a procedure rarely (if ever) seen in a hospital. Guidance provided to accomplish this procedure is empiric (21,22). Similarly, empiric approaches have been developed for home wound care. Surgical site infection should rarely, if ever, be a home-care acquired infection if the wound is primarily closed and no drains are left in place. However, if a surgical patient is sent home with drains, a surgical site infection may develop, and wound-care procedures must address this risk. More frequently, home-care patients have other types of wounds, such as stasis ulcers and pressure sores, which are commonly colonized with gram-negative flora and may become infected with the patient's own organisms. Again, procedures for care of these wounds must be based on the genuine potential for contamination and infection. Arbitrary instructions to discard irrigation fluids at set intervals (e.g., every 24 or 48 hours) are not helpful. Procedures must be practical, with guidance to use containers of fluid that will be used up in two to three visits (i.e., no more than a 500-mL bottle) and incorporate methods to avoid contamination of fluids (e.g., proper handling of the cap, storage away from children and pets) (22).

Many home-care patients receive enteral therapy, introducing the risk for gastrointestinal infection. Again, to reduce this risk, focus must be placed on refrigeration of the enteral feeding and meticulous care of kitchen appliances and tools, such as blenders, used in its preparation. Cleaning blender parts, measuring cups, and spoons in a dishwasher after use is probably sufficient; sterilizing them is probably not necessary (22).

### Use of Barrier Precautions

The rationale and strategy for use of precautions in home care differ substantially from those applied in hospitals (29). In most cases, the use of gowns, gloves, and masks in the care of homebound patients is recommended to protect the health-care provider, not the patient. In addition to standard precautions, care givers in the home may need to use masks only when caring for patients with pulmonary tuberculosis. The exception to this rule may be the home-care patient who is colonized or infected with multidrug-resistant organisms (16,30). Although these organisms are not known to be a risk to providers, they may be transmitted to other home-care patients through inanimate objects or hands. Thus, home-care patients known to have a multidrug-resistant organism should be cared for through use of appropriate barriers. Reusable equipment such as stethoscopes and blood pressure cuffs should remain in the home. If practical, such patients should be seen as the last appointment of the day. If this is not possible, visits should be scheduled to avoid seeing patients at risk, such as those requiring wound care, after seeing a patient with multidrug-resistant organisms.

### The Future of Infection Control in Home Care

The next several years will be critical for developing surveillance systems for home care. Additional studies and reports are needed to improve knowledge of the risk factors for home-care acquired infections. We also need to study the effects of the current empiric practices for preventing such infections. Hospital-based infection control professionals must support and guide their home-care colleagues to develop an evidence-based approach to infection control in home care. A scientific approach will help identify valid risks and successful risk-reduction strategies, as well as improve the quality of care and preserve resources.

Ms. Rhinehart, vice president of quality management for AIG Consultants, Inc., is a full-time health-care consultant. She is one of the principal authors of the revision of CDC's Guidelines for Isolation Precautions in Hospitals (in progress), which will be more applicable to home-care and other ambulatory-care settings.

### References

1. Basic statistics about home care. Washington: National Association for Home Care; 1999.
2. White MC, Ragland KE. Surveillance of intravenous catheter-related infections among home care clients. Am J Infect Control 1994;22:213-35.
3. White MC, Ragland KE. Urinary catheter-related infections among home care patients. Journal of Wound, Ostomy and Continence Nursing 1995;22:286-90.
4. Rosenheimer L. Establishing a surveillance system for infections acquired in home healthcare. Home Healthcare Nurse 1995;13:20-6.
5. Zimay DL. Standardizing the definition and measurement of catheter-related infection in home care: a proposed outcome measurement system. J Med Syst 1999;23:189-99.
6. Luehm D, Fauerbach L. Task force studies infection rates, surgical site management and Foley catheter infections. Caring 1999;18:30-4.
7. Woomer N, Long C, Anderson CO, Greenberg EA. Benchmarking in home health care: a collaborative approach. Caring 1999;18:22-8.
8. Rosenheimer L, Embry FC, Sanford J, Silver SR. Infection surveillance in home care: device-related incidence rates. Am J Infect Control 1998;26:359-63.
9. Goldberg P, Lange M. Development of an infection surveillance project for home healthcare. Home Care Magazine 1997;1:1,4-9.
10. Rhinehart E. Developing an infection surveillance system. Caring 1996;15:26-8, 31-2.
11. Danzig L, Short L, Collins K, Mahoney M, Sepe S, Bland L, et al. Bloodstream infections associated with a needleless intravenous infusion system in patients receiving home infusion therapy. JAMA 23;1995:1862-4.
12. Kellerman S, Shay D, Howard J, Goes C, Feusner J, Rosenberg J, et al. Bloodstream infections in home infusion patients: the influence of race and needleless intravascular access devices. J Pediatr 1996;129:711-7.
13. Tokars JI, Cookson ST, McArthur MA, Boyer CL, McGeer AJ, Jarvis WR. Prospective evaluation of risk factors for bloodstream infections in patients receiving home infusion therapy. Ann Intern Med 1999;131:340-7.
14. Do AN, Ray BJ, Banerjee SN, Illian AF, Barnett BJ, Pham MH, et al. Bloodstream infection associated with needleless device use and the importance of infection-control practices in the home health care setting. J Infect Dis 1999;179:442-8.
15. Friedman M, Rhinehart E. Putting infection control principles into practice in home care. Nurs Clin North Am 1999:34:463-82.
16. Friedman M. Preventing and controlling the transmission of antibiotic-resistant microorganisms in the home care setting. Caring 1999:18:6-11.
17. Davis PL, Madigan EA. Evidence-based practice and the home care nurse's bag. Home Healthcare Nurse 1999;17:295-9.
18. Hanchett M. Implementing standard precautions in home care. Home Care Manager 1998;2:16-20.
19. Friedman M. Designing an infection control to meet JCAHO standards. Caring 1996;15:18-25.
20. Smith PW, Roccaforte JS. Epidemiology and prevention of infections in home healthcare. In: Mayhall CG, editor. Hospital epidemiology and infection control. 2nd ed. Philadelphia: Lippincott Williams and Wilkins; 1999. p. 1483-8.
21. Garofalo K. Home health. In: Olmsted R, editor. APIC infection control and applied epidemiology. St. Louis: Mosby; 1996. p. 90-1–90-11.
22. Rhinehart E, Friedman M. Infection control in home care. Gaithersburg (MD): Aspen Publishers, Inc.; 1999.
23. Garner J, Jarvis WR, Emori TG, Horan T, Hughes J. CDC definitions for nosocomial infections. Am J Infect Control 1988;16:28-40.
24. Emori TG, Culver D, Horan T. National Nosocomial Infections Surveillance System (NNIS): Description of surveillance methods. Am J Infect Control 1991;19:259-67.
25. APIC Home Care Membership Section. Draft definitions for surveillance of infections in home health care. Am J Infect Control 2000;28:449-53.
26. Lee T, Baker O, Lee J, Scheckler W, Steele L, Laxton C. Recommended practices for surveillance. Am J Infect Control 1998:26:277-88.
27. Pearson M. Guideline for prevention of intravascular device-related infections. Infect Control Hosp Epidemiol 1996;17:438-73.
28. Wong ES. Guideline for prevention of urinary tract infection. Am J Infect Control 1983;11:28-31.
29. Garner J. Guideline for isolation precautions in hospitals. Am J Infect Control 1996;17:53-80.
30. Centers for Disease Control and Prevention. Recommendations for preventing the spread of vancomycin resistance. Am J Infect Control 1995;23:87-94.
